# Modification of platinum sensitivity by KEAP1/NRF2 signals in non-small cell lung cancer

**DOI:** 10.1186/s13045-016-0311-0

**Published:** 2016-09-06

**Authors:** Yijun Tian, Kongming Wu, Qian Liu, Na Han, Li Zhang, Qian Chu, Yuan Chen

**Affiliations:** Department of Oncology, Tongji Hospital of Tongji Medical College, Huazhong University of Science and Technology, 1095 Jiefang Avenue, Wuhan, 430030 China

**Keywords:** NRF2, KEAP1, Non-small cell lung cancer, HO1, NQO1, GCLM, Nedaplatin, Cisplatin

## Abstract

**Background:**

The objective of this study was to evaluate the effect of platinum-based drugs on nuclear-factor erythroid2 like 2 (NRF2) signaling in non-small cell lung cancer cell lines with or without Kelch-like ECH-associated protein 1 (KEAP1) mutations and to determine the role of NRF2 and KEAP1 on platinum-based drug treatment.

**Methods:**

We used real-time PCR to assess relative mRNA expression and used western blotting and immunofluorescence assays to assess protein expression. Small interfering RNA and shuttle plasmids were used to modulate the expression of NRF2, wild-type KEAP1, and mutant KEAP1. Drug sensitivity to platinum-based drugs was evaluated with Cell Count Kit-8.

**Results:**

We found that platinum-based therapies modified the NRF2 signaling pathway differently in KEAP1-mutated non-small cell lung cancer (NSCLC) cell lines compared with wild-type KEAP1 cell lines. The reactive degree of NRF2 signaling also varies between nedaplatin and cisplatin. The modification of NRF2 or KEAP1 expression in NSCLC cell lines disrupted downstream gene expression and cell sensitivity to platinum-based drugs. Finally, gene expression data retrieved from The Cancer Genome Atlas (TCGA) consortium indicated that KEAP1 mutation significantly affects NRF2 signaling activity in patients with NSCLC.

**Conclusions:**

Our findings suggest that NRF2 signaling plays an indispensable role in NSCLC cell sensitivity to platinum-based treatments and provides a rationale for using NRF2 as a specific biomarker for predicting which patients will be most likely to benefit from platinum-based treatment.

## Background

Non-small cell lung cancer (NSCLC) is associated with considerably high mortality worldwide [1]. Surgery can ensure local disease control in patients with early stages of disease, and patients unsuitable for surgery at diagnosis, primarily those with advanced stage disease, receive chemotherapy or targeted therapy as a first line of treatment. Epidermal growth factor receptor tyrosine kinase inhibitor (EGFR-TKI) and echinoderm microtubule-associated protein-like 4-anaplastic lymphoma kinase fusion tyrosine kinase inhibitor (EML4-ALK-TKI) exert substantial therapeutic effects in lung adenocarcinomas with specific commonly occurring mutations [2–5]. Platinum-based treatment regimens remain the standard of care recommended by the lung cancer guidelines for squamous lung cancer or adenocarcinoma with undefined mutation status [6]. Recently, studies [7, 8] have revealed that a nedaplatin-based treatment regimen is associated with a favorable response in patients with squamous cell lung cancer. Platinum-based drugs are defined as DNA adduct-forming agents that exert antineoplastic effects by inducing the formation of double-stranded DNA cross-links, thus resulting in irreversible DNA damage [9]. Accumulating DNA damage eventually leads to apoptosis [10, 11].

Nuclear-factor (NF)-E2-related factor 2 (NRF2) is a core transcription factor involved in the cellular response to oxidative stress [12]. Under basal conditions, NRF2 forms a complex with Kelch-like ECH-associated protein-1 (KEAP1), which is targeted for ubiquitination. Upon exposure to stress or electrophilic regents, NRF2 evades the ubiquitination process and translocates from the cytoplasm to the nucleus [13]. By binding to antioxidant responsive element (ARE), NRF2 increases the transcription of multiple cytoprotective enzymes [14] such as heme oxygenase-1(HO1), NAD (P) H dehydrogenase (quinone) 1 (NQO1), and glutamate-cysteine ligase modifier (GCLM) [15].

In urethane-induced lung carcinogenesis model, NRF2 activation suppresses tumorigenesis, but somatic activation of NRF2 in cancer cells enhanced growth of tumors [16]. Studies have demonstrated that NRF2 activation contributes to chemo-resistance [17–19] or radio-resistance [20] in lung cancers. Investigators have also focused on the relationship between NRF2 activity and platinum treatment in NSCLC cell line [21, 22]. However, studies designed to observe and compare NRF2 signaling response under different platinum treatment are rarely reported [23]. In this study, we sought to elucidate the role of NRF2 in platinum-based chemotherapy for NSCLC, and we identified KEAP1 mutation as a key factor contributing to platinum sensitivity.

## Methods

### Chemicals and cell culture

Unless otherwise stated, all chemicals were purchased from Sigma-Aldrich (Shanghai, China). Antibodies against NRF2 (ab62352) were purchased from Abcam; antibodies against HO1 (#5853) and NQO1 (#3187) were purchased from Cell Signaling Technology; and antibodies against KEAP1 (10503-2-AP), GCLM  (14241-1-AP), and β-actin were purchased from Proteintech.

The A549 (human lung adenocarcinoma), H292 (human mucoepidermoid carcinoma), H460 (human large cell lung cancer), and SKMES-1 (human squamous lung cancer) cell lines were obtained from the Cell Resource Center, Peking Union Medical College. The cells were cultured in RPMI-1640, with the exception of SKMES-1 cells, which were cultured in MEM. All media were supplemented with 10 % fetal bovine serum (GIBCO, NY, USA). No antibiotics were added to the media.

### Plasmid and siRNA design

The wild-type KEAP1 coding sequence (CDS) was cloned into the pEnter shuttle plasmid. The Fast Mutagenesis system (Transgene, Beijing, China) was used to introduce the G333C and D236H mutations. The primers used were:

- G333C-forward, TGATCTACACCGCGGGCTGCTACTTCCGACAGTC
- G333C-reverse, AGCCCGCGGTGTAGATCAGGCGGCCCACC
- D236H-forward, AGCCCGCGGTGTAGATCAGGCGGCCCACC
- D236H-reverse, GGTCCCGGCTGATGAGGGTCACCAGTTGG
- The small interfering RNAs used to silence NRF2 were:
- Sense, GGUUGAGACUACCAUGGUUTT
- Antisense, AACCAUGGUAGUCUCAACCTT

Transfection was conducted with Lipofectamine 2000 (Invitrogen) according to a previously published method [24].

### Real-time PCR analysis

Total messenger RNA (mRNA) was extracted from cells using TRIzol according to the manufacturer’s protocol (Takara, Japan). Seven hundred fifty-nanogram total mRNA was used to synthesize first strand complementary DNA (cDNA) using the cDNA synthesis kit (ThermoFisher Scientific). Quantification reactions were performed with the ABI 7900 HT platform. Each reaction consisted of 5 μl SYBR Green/ROX qPCR Master Mix (ThermoFisher Scientific), 1 μl cDNA, 1 μl 10 μM forward and reverse primer mix, and 3 μl ddH_2_O. Cycling conditions were as follows: 95 °C for 10 min and 40 cycles of 95 °C for 15 s and 60 °C for 60 s. Cycle thresholds were calculated as the expression fold-changes according to the 2^−△△CT^ algorithm. The primers used for real-time PCR are:

- NRF2, forward, TCCAGTCAGAAACCAGTGGAT
- Reverse, GAATGTCTGCGCCAAAAGCTG
- HO1, forward, AAGATTGCCCAGAAAGCCCTGGAC
- Reverse, AACTGTCGCCACCAGAAAGCTGAG
- NQO1, forward, GAAGAGCACTGATCGTACTGGC
- Reverse, GGATACTGAAAGTTCGCAGGG
- GCLM, forward, TGTCTTGGAATGCACTGTATCTC
- Reverse, CCCAGTAAGGCTGTAAATGCTC

### KEAP1 mutation sequencing

Total RNA was extracted and used to synthesize the first chain cDNA according to the procedure described in the real-time PCR analysis description. cDNA was used as a template to synthesize six segments of the KEAP1 gene using the following cycling conditions: initial denaturing step at 94 °C for 5 min followed by 30 cycles at 94 °C for 40 s, 60 °C for 40 s, and 72 °C for 55 s. Products were separated on 1 % agarose gels, and bands were visualized with ethidium bromide. The products were sequenced using the ABI3730 XL DNA Analyzer (Applied Biosystem Japan, Tokyo, Japan) and analyzed with Chromas 2.4.3 software. Primers used to amplify the KEAP1 gene fragments were:

- Fragment 1, forward AGAGGTGGTGGTGTTGCTTAT
- Reverse TGGAGATGGAGGCCGTGTA
- Fragment 2, forward CAGGTCAAGTACCAGGATG
- Reverse GATGAGGGTCACCAGTTG
- Fragment 3, forward ATCGGCATCGCCAACTTC
- Reverse AGGTAGCTGAGCGACTGT
- Fragment 4, forward CAGAAGTGCGAGATCCTG
- Reverse GCTCTGGCTCATACCTCT
- Fragment 5, forward GCCCTGGACTGTTACAAC
- Reverse GTCTCTGTTTCCACATCGTA
- Fragment 6, forward GCTGTCCTCAATCGTCTC
- Reverse AGTTCTGCTGGTCAATCTG
- NRF2 exon2, forward TCGTGATGGACTTGGAGCTG
- Reverse AGCATCTGATTTGGGAATGTG

Sections were spliced together after manual inspection and compared with the reference sequence with BLAST to identify potential mutations.

### Western blot analysis

Cytoplasm and nuclear protein were extracted by using NE-PER™ Nuclear and Cytoplasmic Extraction Reagents (ThermoFisher Scientific). For total protein extraction, we used standard protocols for protein isolation and antibody detection, as previously described [24, 25]. Cells were washed twice with ice-cold PBS and then lysed with RIPA buffer. Each sample was denatured in 100 °C boiling water in the presence of SDS and DTT. A total of 20 μg of protein from each sample was loaded onto a 10 % SDS-PAGE gel for separation by electrophoresis. A tank system was used to transfer proteins from gels to PVDF membranes. The membranes were blocked with nonfat milk for 1 h and incubated with primary antibodies overnight. All primary antibodies were used at a dilution of 1:1000, with the exception of anti-β-actin (1:5000) and anti-NQO1 (1:3000). Blots were sufficiently washed in 0.1 % Tris-buffered saline and Tween20 (TBST) four to six times for at least 5 min per wash. Washed membranes were incubated with secondary antibodies at a dilution of 1:5000 for 1 h at room temperature. After incubation with secondary antibodies, the membranes were washed three times for at least 5 min per wash. SuperSignal West Pico Chemiluminescent Substrate (ThermoFisher Scientific) was applied for exposure using Syngene G: BOX F3 Fluorescence Imaging System. All images of the blots were processed in Photoshop without gray value modification and spliced in Microsoft PowerPoint.

### Immunofluorescence staining

Immunofluorescence was performed as previously reported with some modifications [26]. Cells were seeded onto a 24-well plate until they reached a confluence of 50~60 % and were treated with the indicated reagents. After treatment, cells were fixed in 4 % paraformaldehyde at 4 °C for 10 min. The cells were then washed in PBS for 5 min and permeabilized and blocked with 0.1 % Triton X-100 and 1 % bovine serum albumin PBS for 20 min. Cells were incubated with primary antibody against HO1 at a dilution of 1:200 at 4 °C overnight. The cells were then washed in PBS three times for 5 min per wash. Cells were incubated with Alexa Fluor 488® secondary antibody (Invitrogen) at a dilution of 1:1000 for 1 h at room temperature. DAPI solution was added after incubation with secondary antibody. Cells were washed three times for 5 min per wash before imaging. Fluorescence images were captured using a Leica digital microscope (Leica Microsystems, Wetzler, Germany). Images were merged using Adobe Photoshop CS6, and no other modifications were made.

### Cytotoxic assay

Cellular proliferation was evaluated using a Cell Count Kit-8 (CCK-8) with some modifications [27]. Lung cancer cell lines were seeded onto 96-well plates for attachment for a minimum of 12 h in a 37 °C incubator. Nedaplatin or cisplatin dissolved in phosphate-buffered saline (PBS) was added to the medium at a concentration gradient of 30–150 μM. After 24 h of exposure, the medium was completely removed. Then, a medium with 10 % (*v*/*v*) CCK-8 was added to each well. Absorbance at 490 nm was measured after a 2-h incubation at 37 °C. Cell survival was calculated as the ratio between the treated group and control groups. Ratios were graphed in line charts with Graphpad Prism 6 software.

### Public database analysis

Expression data from The Cancer Genome Atlas (TCGA) squamous lung cancer patients was retrieved using the package TCGA-Assembler (http://www.compgenome.org/TCGA-Assembler/) in an R program. KEAP1 mutation status was defined as previously described [28]. Specimens with a confirmed KEAP1 mutation status were included for evaluation of relative gene expression values.

### Statistical methods

Parametric tests were used to evaluate data with a normal distribution. Non-parametric tests were used to evaluate data with unknown distribution patterns. mRNA fold-changes were compared using Student’s *t* tests. Survival comparisons were conducted using paired *t* tests. Comparisons between the mutant and wild-type KEAP1 groups were conducted using Mann-Whitney tests. Significance was set at *P* < 0.05.

## Results

### KEAP1 mutation status influenced the expression of NRF2 downstream target genes in cells exposed to platinum-based treatment

**Fig. 1:**
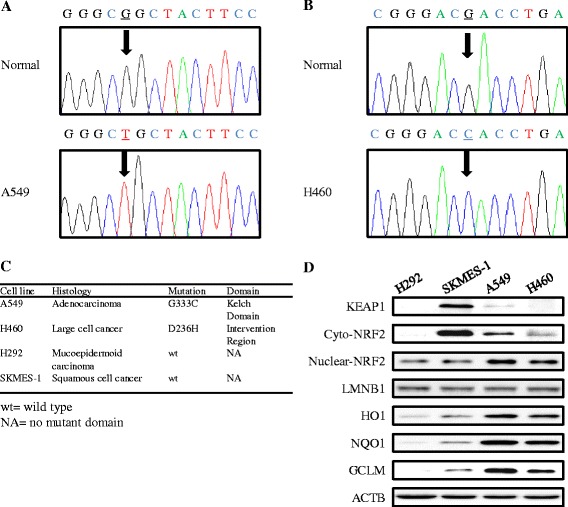
Identification of KEAP1 mutations and basal expression levels of NRF2-regulated genes in NSCLC. Sequencing of the KEAP1 gene led to the identification of G333C mutation in A549 cells (**a**) and the D236H mutation in H460 cells (**b**). The *arrow* indicates the mutant peak. **c** Characteristics of the four NSCLC cell lines and a description of the KEAP1 mutations. NA, no mutant domain. **d** Basal expression of KEAP1, NRF2, and NRF2-regulated genes in the four NSCLC cell lines

KEAP1 is a key regulator of NRF2 function, and the role of wild-type KEAP1 in negatively regulating NRF2 signaling has been well characterized. We first sequenced the KEAP1 gene in four NSCLC cell lines to identify potential mutations and found that the A549 cell line harbors a G333C mutation within the first Kelch domain (KLD) (Fig. 1a) and that the H460 cell line harbors a D236H mutation in the intervention region (IVR) (Fig. 1b). No KEAP1 mutations were identified in the H292 and SKMES-1 cell lines. Sequencing of NRF2 exon 2 in the four NSCLC cell lines demonstrated that no mutations existed in this locus. We then evaluated basal protein levels of KEAP1 and components of the NRF2 signaling pathway in the four NSCLC cell lines. NRF2 protein in nuclear extracts and the proteins encoded by the three genes downstream of NRF2, HO1, NQO1, and GCLM were more highly expressed in A549 and H460 cells than in H292 and SKMES-1 cells (Fig. 1d).

**Fig. 2:**
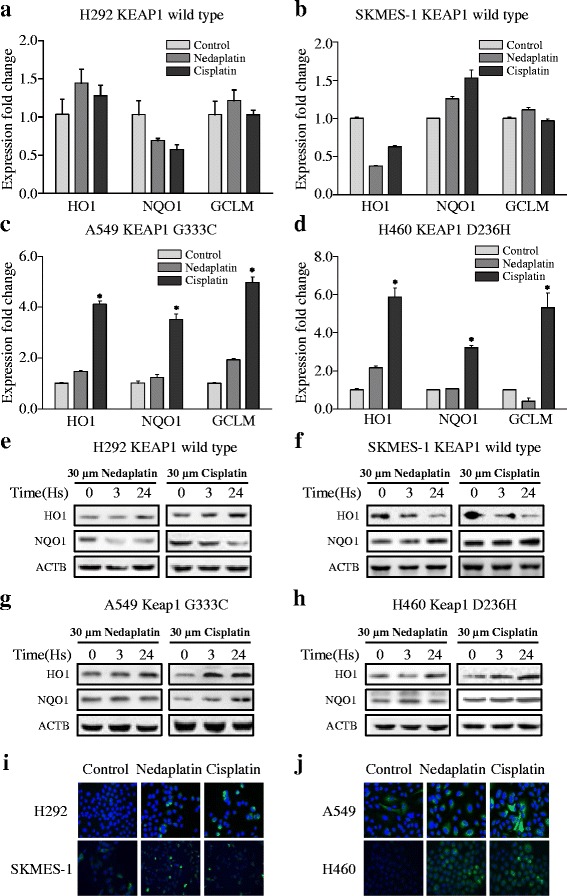
Comparison of NRF2-regulated gene expression in NSCLC cells exposed to platinum-based drugs. **a**, **b** The levels of HO1, NQO1, and GCLM mRNA expression in H292 and SKMES-1 cells exposed to 30 μM nedaplatin or cisplatin for 24 h. **c**, **d** The expression levels of HO1, NQO1, and GCLM mRNA in A549 and SKMES-1 cells exposed to 30 μM nedaplatin or cisplatin for 24 h. Comparison was conducted between 24-h points and control group by using Student’s tests. The results are presented as the mean ± SEM. *p < 0.01, **p < 0.05. **e**, **f** The levels of HO1, NQO1, and GCLM protein expression in H292 and SKMES-1 cells exposed to 30 μM nedaplatin or cisplatin for 3 or 24 h. **g**, **h** The levels of HO1, NQO1, and GCLM protein expression in A549 and H460 cells exposed to 30 μM nedaplatin or cisplatin for 3 or 24 h. **i** Immunofluorescence staining of HO1 in H292 (*upper panel*) and SKMES-1 (*lower panel*) cells exposed to 30 μM nedaplatin or cisplatin over a 24-h treatment period. **j** Immunofluorescence staining of HO1 in H460 (*upper panel*) and A549 (*lower panel*) exposed to 30 μM nedaplatin or cisplatin over a 24-h treatment period

To profile the expression of NRF2 signaling pathway components in response to platinum treatment, the four NSCLC cell lines (H292, SKMES-1, A549, and H460) were treated with 30 μM nedaplatin or 30 μM cisplatin for 24 h. Total RNA from each group was extracted, and HO1, NQO1, and GCLM gene expression levels were assessed with real-time PCR. In the wild-type KEAP1 cell lines H292 (Fig. 2a) and SKMES-1 (Fig. 2b), nedaplatin and cisplatin slightly enhanced the expression of NRF2 downstream target genes after 24 h of treatment. In the mutant KEAP1 cell line A549 (Fig. 2c, *p < 0.01, **p < 0.05) and H460 (Fig. 2d, *p < 0.01, **p < 0.05), nedaplatin induced a weak but significant increase in three downstream gene expression. Cisplatin treatment, however, significantly enhanced HO1, NQO1, and GCLM expression in both A549 and H460 cell lines after 24 h of treatment.

To evaluate protein levels, the four cell lines were treated with 30 μM nedaplatin or 30 μM cisplatin for 3 or 24 h. Total proteins were extracted, and HO1, NQO1, GCLM protein expression was evaluated by using western blot analysis. In the wild-type KEAP1 H292 and SKMES-1 cell lines (Fig. 2e, f), a mild increase or no change of NQO1 and HO1 protein was observed after 3- or 24-h treatment with either platinum-based drug. In the mutant KEAP1 cell lines A549 (Fig. 2g) and H460 (Fig. 2h), a mild increase or no change was observed in NQO1 and HO1 protein levels after nedaplatin treatment, whereas the expression of these proteins was substantially enhanced after 24 h of treatment with cisplatin.

The results of immunofluorescence analysis of HO1 protein expression were consistent with the results of the western blot analysis. Over a 24-h treatment period, 30 μM nedaplatin or cisplatin induced a mild increase in HO1 protein expression in H292 and SKMES-1 cells (Fig. 2i). In the A549 and H460 cell lines (Fig. 2j), both platinum-based drugs enhanced the expression of HO1 to a greater degree over the same time period.

### KEAP1 mutations modified NRF2 signaling and influenced platinum sensitivity

**Fig. 3:**
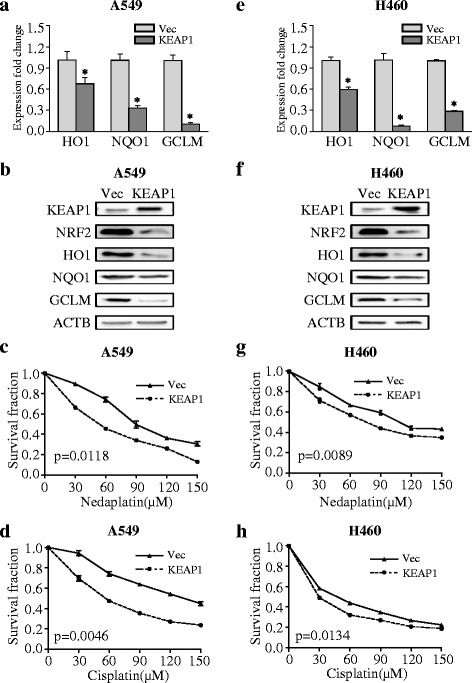
KEAP1 overexpression inhibits NRF2 signaling and increases chemosensitivity to nedaplatin or cisplatin in KEAP1-mutant cells. **a**, **b** mRNA and protein levels of NRF2-regulated genes in A549 cells overexpressing wild-type KEAP1. **c**, **d** KEAP1 overexpression modified the sensitivity of A549 cells to platinum-based drugs. **e**, **f** mRNA and protein levels of NRF2 downstream genes in H460 cells overexpressing wild-type KEAP1. **g**, **h** KEAP1 overexpression modified the sensitivity of H460 cells to platinum-based drugs. The results are presented as the mean ± SEM. *p < 0.001, **p < 0.05. *Vec* vector

To investigate the impact of KEAP1 mutations status on NRF2 signaling, the KEAP1 CDS was cloned into the pEnter vector and transfected into the NSCLC cell lines. In the mutant KEAP1 cell lines A549 (Fig. 3a, b, *p < 0.001, **p < 0.05) and H460 (Fig. 3e, f, *p < 0.001), constitutive expression of wild-type KEAP1 over a 72-h period reduced the expression of the NRF2 downstream target genes HO1, NQO1, and GCLM at the mRNA and protein levels. Overexpression of wild-type KEAP1 sensitized A549 cells (Fig. 3c, d, paired *t* test *p* = 0.0118 for nedaplatin, paired *t* test *p* = 0.0046 for cisplatin) and H460 cells (Fig. 3g, h, paired *t* test *p* = 0.0089 for nedaplatin, paired *t* test *p* = 0.0134 for cisplatin) to nedaplatin and cisplatin over a 24-h treatment period.

**Fig. 4:**
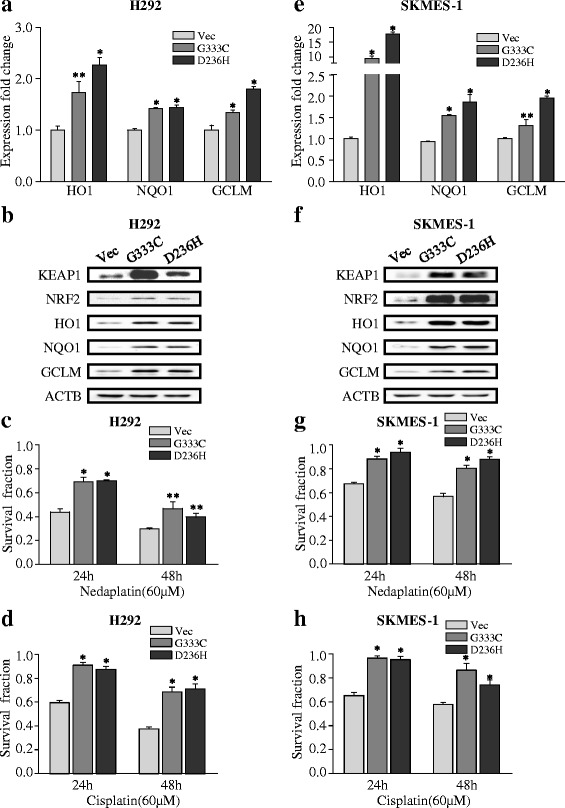
KEAP1 mutations activate NRF2 signaling and confer chemo-resistance. **a**, **b** KEAP1 with the G333C or D236H mutations induced the expression of NRF2 downstream target genes at the mRNA and protein levels in H292 cells. **c**, **d** KEAP1 with the G333C or D236H mutations increased cell survival in H292 cells exposed to 60-μm platinum-based drugs. **e**, **f** KEAP1 with the G333C or D236H mutation induced the expression of NRF2-associated genes at the mRNA and protein levels in SKMES-1 cells. **g**, **h** KEAP1 with the G333C or D236H mutation increased cell survival in SKMES-1 cells exposed to 60-μm platinum-based drugs. The results are presented as the mean ± SEM. *p < 0.001, **p < 0.05. *Vec* vector

Next, we introduced the G333C and D236H mutations into the KEAP1 pEnter construct. The mRNA expression levels of the NRF2-associated genes HO1, NQO1, and GCLM were significantly elevated in H292 cells (Fig. 4a, *p < 0.001, **p < 0.05) and SKMES-1 cells (Fig. 4e, *p < 0.001, **p < 0.05) transfected with the mutant KEAP1 construct. An identical trend was observed at the protein level (Fig. 4b, f). Transfection of the mutant KEAP1 plasmid significantly increased survival in H292 cells (Fig. 4c, d, *p < 0.001, **p < 0.05) and SKMES-1 (Fig. 4g, h, *p < 0.001) treated with 60 μM cisplatin or nedaplatin.

### NRF2 activity altered downstream gene expression and influenced platinum sensitivity

**Fig. 5:**
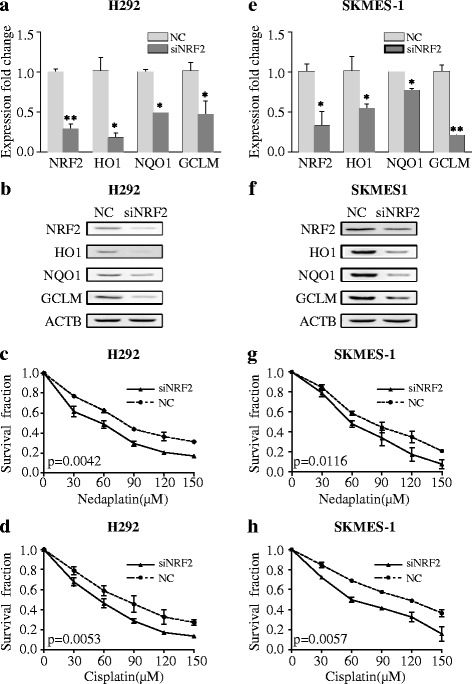
siRNA inhibited NRF2 signaling and increased chemosensitivity to platinum-based drugs. **a**, **b** siRNA knockdown of NRF2 reduced the expression of NRF2 and NRF2-regulated genes at the mRNA and protein levels in H292 cells. **c**, **d** siRNA increased the sensitivity of H292 cells to platinum-based drugs. **e**, **f** siRNA targeting NRF2 reduced the expression of NRF2 and NRF2-associated genes at the mRNA and protein levels in SKMES-1 cells. **g**, **h** siRNA knockdown of NRF2 increased platinum sensitivity in SKMES-1 cells. The results are presented as the mean ± SEM. *p < 0.001, **p < 0.05. *Vec* vector

To assess the effects of NRF2 expression on NSCLC cells exposed to platinum-based drugs, we designed a small interfering RNA (siRNA) construct targeting NRF2 mRNA to inhibit NRF2 activity. In the wild-type KEAP1 cell lines H292 (Fig. 5a, b, *p < 0.001, **p < 0.05) and SKMES-1 (Fig. 5e, f, *p < 0.001, **p < 0.05), siRNA transfection over a 72-h period reduced the expression of NRF2 and its downstream targets HO1, NQO1, and GCLM at the mRNA and protein levels. Pretreatment with siRNA targeting NRF2 increased sensitivity to nedaplatin and cisplatin in H292 cells (Fig. 5c, d, paired *t* test *p* = 0.0042 for nedaplatin, paired *t* test *p* = 0.0053 for cisplatin) and SKMES-1 (Fig. 5g, h, paired *t* test *p* = 0.0116 for nedaplatin, paired *t* test *p* = 0.0057 for cisplatin) over a 24-h treatment period.

**Fig. 6:**
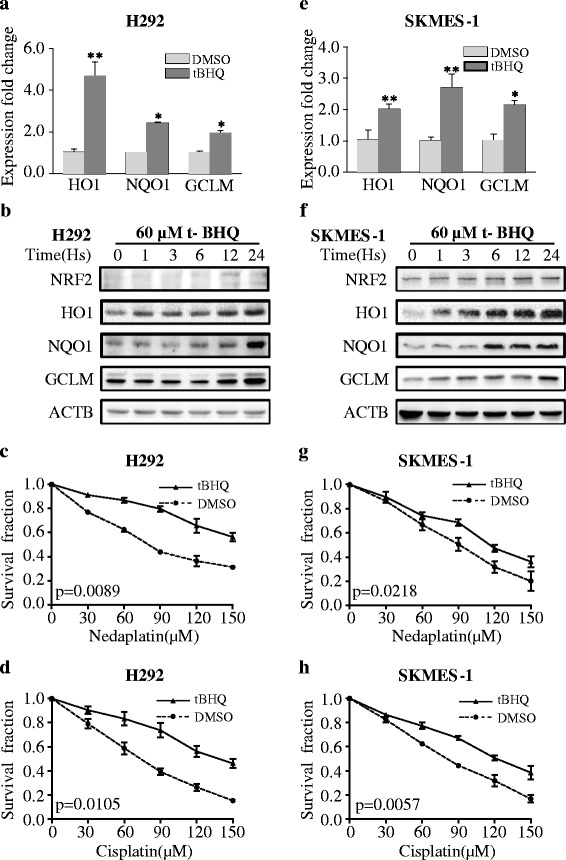
t-BHQ activated NRF2 signaling and conferred chemo-resistance to platinum-based drugs. **a**, **b** t-BHQ induced the expression of NRF2 and NRF2-regulated genes at the mRNA and protein levels in H292 cells. **c**, **d** t-BHQ conferred chemo-resistance to platinum-based drugs in H292 cells. **e**, **f** t-BHQ induced the expression of NRF2 and NRF2-regulated genes at the mRNA and protein levels in SKMES-1 cells. **g**, **h** t-BHQ conferred chemo-resistance to platinum-based drugs in SKMES-1 cells. The results are presented as the mean ± SEM. *p < 0.001, **p < 0.05. *Vec* vector

In addition, NSCLC cell lines were treated with t-BHQ, a specific activator of NRF2. In the wild-type KEAP1 NSCLC cell lines H292 (Fig. 6a, b, *p < 0.001, **p < 0.05) and SKMES-1 (Fig. 6e, f, *p < 0.001, **p < 0.05), administration of the NRF2 activator over a 24-h period significantly increased the expression of the NRF2 downstream genes HO1, NQO1, and GCLM at the mRNA and protein levels. Pretreatment with 60 μm t-BHQ conferred chemo-resistance to nedaplatin and cisplatin in H292 cells (Fig. 6c, d, paired *t* test *p* = 0.0089 for nedaplatin, paired *t* test *p* = 0.0105 for cisplatin) and in SKMES-1 cells (Fig. 6g, h, paired *t* test *p* = 0.0218 for nedaplatin, paired *t* test *p* = 0.0057 for cisplatin) over a 24-h treatment period.

### KEAP1 mutation enhanced NRF2 signals expression in NSCLC patients

**Fig. 7:**
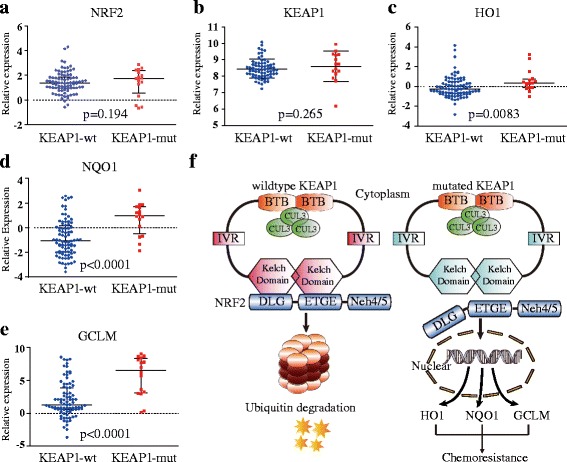
Comparison of NRF2-associated genes mRNA between KEAP1-wt and KEAP1-mut cases in squamous lung cancer. **a** NRF2 mRNA expression levels in KEAP1-wt and KEAP1-mut cases. **b** KEAP1 mRNA expression levels in KEAP1-wt and KEAP1-mut cases. **c** HO1 mRNA levels between KEAP1-wt and KEAP1-mut cases. **d** NQO1 mRNA levels in KEAP1-wt and KEAP1-mut cases. **e** GCLM mRNA expression levels in KEAP1-wt and KEAP1-mut cases. **f** Schematic diagram of wild-type (*left*) and mutant KEAP1 protein (*right*) function. Mutant KEAP1 was predicted to exhibit an attenuated unity. The increased nuclear accumulation and transcriptional activity of NRF2 upregulate the expression of cytoprotective genes such as HO1, NQO1, and GCLM, thereby leading to cancer cell chemo-resistance. *BTB*, broad complex, tramtrack, and Bric à brac, *IVR* intervention region, *wt* wild type, *mut* mutation. The results are presented as the median ± quartile

A total of 100 individuals diagnosed with NSCLC were selected from the TCGA group for analysis. KEAP1 mutation status was defined as previously described [28]. We found that 86 patients harbored the wild-type KEAP1 allele, and 16 patients harbored a KEAP1 mutation. Gene expression data from patients with mutant or wild-type KEAP1 were used to generate a scatter plot diagram. No significant differences in the mRNA expression levels of NRF2 (Fig. 7a Mann-Whitney test *p* = 0.193) and KEAP1 (Fig. 7b Mann-Whitney test *p* = 0.265) were observed between the mutated patients with mutant KEAP1 cases and with wild-type KEAP1. However, expression levels of the NRF2 downstream target genes HO1 (Fig. 7c Mann-Whitney test *p* = 0.0083), NQO1 (Fig. 7d Mann-Whitney test *p* < 0.0001), and GCLM (Fig. 7e Mann-Whitney test *p* < 0.0001) were significantly enhanced in patients with mutant KEAP1 cases compared with those with wild-type KEAP1.

## Discussion

A large genomic study has demonstrated that multiple oncogenic and tumor suppressor pathways are involved in the initiation and progression of lung cancer [29]. In addition to the well-known oncogenic pathways, such as those mediated by retrovirus-associated DNA sequences (RAS), epidermal growth factor receptor (EGFR), and anaplastic lymphoma kinase (ALK), novel signaling pathways continue to be identified, such as those mediated by NOTCH [30, 31] and DACH1 [26, 32]. Targeted drugs already serve as a first-line therapy for NSCLC patients with specific mutations. However, for the many NSCLC patients with an unknown mutation status or with no gene mutations, platinum-based regimens remain the standard of care proposed by NCCN guideline. Previous studies [20, 33] have revealed that NRF2 and its downstream genes play pivotal protective roles in NSCLC chemotherapy. A recent genomic analysis has revealed that disruptions in the KEAP1/NRF2 pathway are observed in nearly 30 % of squamous lung cancer patients [28]. KEAP1 mutations were more common in smoked NSCLC. Research by Takahashi et al. [34] demonstrated all four KEAP1 mutated lung cancer patients were heavy smoker with a mean pack year value of 79.0, while no KEAP1 mutation was detected in no-smoked patients. Platinum-induced DNA damage and cellular response to stress might be associated with the efficacy of chemotherapy and might contribute to resistance [10, 12, 35].

Functional KEAP1 represses NRF2 activity by recruiting the NRF2 protein to the actin cytoskeleton [36]. Sequencing experiments revealed that the A549 cell line harbors a mutation (glycine 333 to cysteine) in the Kelch domain and that the H460 cell line harbors a mutation (aspartic acid 236 to histidine) in the intervention region of KEAP1. No mutations in exon 2 of the NRF2 gene, the site associated with most NRF2 mutations [37], have been identified in sequencing analysis. We observed increased basal levels of NRF2 downstream target genes in the NSCLC cell lines harboring KEAP1 mutations compared with the wild-type KEAP1 NSCLC cell lines. Interestingly, an increased protein level of cytoplasm NRF2 in squamous cell lung cancer cell line SKMES-1 was observed. But the high cytoplasm NRF2 protein did not lead to increased NRF2 nuclear accumulation and expression of NRF2 downstream target genes, suggesting that NRF2 function is tightly regulated by wild-type KEAP1. Our findings provide a visual profile of the functional and aberrant KEAP1-NRF2 interactions observed in NSCLC.

After exposure to cisplatin, the KEAP1 mutant cell lines A549 and H460 exhibited considerably increased levels of NRF2 signaling, whereas when the KEAP1 wild-type cell lines H292 and SKMES-1 exhibited no changes or only mild changes. Cisplatin is a weak inducer of ARE in the MCF-7 human breast cancer cell line (without KEAP1 mutation [38]) and induces a 1.3-fold change in ARE expression levels after 24 h of exposure [15], consistently with our observations in wild-type KEAP1 NSCLC cells. Interestingly, in KEAP1 mutant cell lines, two platinum drugs induced different level of increasing extents of NRF2 signaling. That is less than 2.5-fold induction for NRF2 by nedaplatin and more than 3.5-folds by cisplatin were observed. This phenomenon was mostly seen on HO1 gene. HO1 blots in Fig. 2g, h demonstrated that nedaplatin induced lower while cisplatin induced higher elevation of HO1 gene in A549 and H460. Reactive oxygen species (ROS) play a role in cisplatin induced and activated by a variety of signals [39, 40]. KEAP1 perceives cellular ROS levels via its multiple amino acid domains [38, 41, 42]. KEAP1 mutations disrupt the interaction between KEAP1 and NRF2 [22]; however, the kinetics of this interaction in KEAP1 mutant cells exposed to cisplatin have not previously been described. Our findings suggest that NRF2 signaling is upregulated in NSCLC cells harboring KEAP1 mutations.

However, nedaplatin induces only weak activation or no activation of NRF2 signaling. The antineoplastic activity of nedaplatin, a cisplatin derivate developed in 1983 [43], might be mediated by mechanisms distinct from those of p53-dependent early apoptosis [44]. Several studies have demonstrated that a nedaplatin-based treatment regimen [45–47] for squamous cell lung cancer is superior to a cisplatin or carboplatin-based regimen [48]. Our work provides a potential rationale for nedaplatin as the optimal choice for NSCLC patients with KEAP1 mutations. Platinum-based drugs are capable of binding DNA [49], explaining why we observed a decrease in the expression of NRF2 downstream genes during early phases (3 h) of platinum exposure in wild-type KEAP1 H292 cell line.

An early study by Devling et al. [50] has revealed that inhibition of KEAP1 function markedly enhances endogenous levels of NRF2. Our results demonstrated that transfection of wild-type KEAP1 potently attenuated NRF2 signaling and sensitized A549 and H460 cells to platinum-based treatment. In addition, expression of G333C and D236H mutant KEAP1 increased the expression of NRF2 downstream genes at the mRNA and protein levels and resulted in increased cell survival after exposure to platinum-based drugs. Most KEAP1 mutations enhance the nuclear localization of NRF2, thereby leading to the constitutive activation of downstream gene expression [51]. Although nedaplatin induced lower activation of NRF2 signal in KEAP1 mutant cell line, its sensitivity can be influenced by intervention on NRF2 activity. As NRF2 upregulate a series of detoxification genes and protect cancer cells against insults, our results demonstrated that the sensitivity of nedaplatin may be influenced by other NRF2 downstream genes, including but not limited to AKR1C3, GST, and PSAT1 [52]. In addition, in Fig. 4c, d, Keap1 mutation in H292 cell line conferred indeed less resistance to nedaplatin than cisplatin. But this superiority for nedaplatin was not that significant in SKMES-1 cells (Fig. 4g, h). The underneath mechanism requires validation. In summary, we demonstrated that KEAP1 mutations influence NRF2 signaling and platinum sensitivity in NSCLC cells.

NRF2 activity is involved in chemosensitivity in breast cancer [20] and ovarian cancer [33]. Clinical evidence [53, 54] has also suggested that NRF2 signaling confers chemo-resistance in NSCLC. We found that siRNA knockdown of NRF2 or NRF2 activation significantly disrupted NRF2 signaling in vitro and led to sensitization or resistance of NSCLC cells to platinum-based drugs. It demonstrated again that NRF2 signals had a significant impact on platinum sensitivity in H292. We expected to see that NRF2 activity have no impact on nedaplatin sensitivity, but repeated experiment demonstrated a minor but significant effect of NRF2 on nedaplatin sensitivity (paired *t* test *p* value 0.0116). This effect was also observed in Fig. 6g (paired *t* test *p* value 0.0218). We speculated that under siRNA intervention or activator treatment, some other pathways such as NF-kappa B and BACH1 are compensatorily involved in the process [55]. Our work provides some new insights into the relationship between NRF2 signaling and platinum-based chemotherapy.

To confirm the significance of KEAP1 mutation in vivo, we evaluated public gene expression data from the publically available TCGA consortium. As expected, KEAP1 mutation did not significantly change alter the mRNA expression of NRF2 and KEAP1. However, mRNA levels of NRF2 downstream target genes were significantly higher in patients with KEAP1 mutations compared with patients with wild-type KEAP1, suggesting that KEAP1 and NRF2 interact primarily at the protein level and that KEAP1 mutations strongly affect NRF2 signaling (Fig. 7f). The association of clinical response with these mutations was not well defined before. One mentioned study evaluated the impact of KEAP1 alteration on NSCLC patients’ survival [34]. In this study, KEAP1 mutation predicted a worse overall survival. As to disease-free survival, this study demonstrated a vague trend toward significance.

## Conclusions

In summary, the mRNA and protein expression profile of NRF2 and components of the NRF2-associated genes in four NSCLC cell lines were characterized and their responses to platinum-based therapies were analyzed. A causative effect of KEAP1-NRF2 signaling on platinum-based treatment was defined by engineered expressing either wild-type or mutant KEAP1 and knocking-down or activating NRF2. It is reasonable to hypothesize that KEAP1/NRF2 plays a key role in the cellular response to platinum chemotherapy in NSCLC and that KEAP1 could be explored as a specific biomarker for predicting a patient’s response in personalized therapy.

## Abbreviations

KEAP1: Kelch-like ECH-associated protein 1
NRF2: Nuclear-factor erythroid2 like 2
NSCLC: Non-small cell lung cancer
BTB: Broad complex, tramtrack, and bric à brac
IVR: Intervention region
wt: Wild type
mut: Mutation
HO1: Heme oxygenase-1
NQO1: NAD (P) H-quinone oxidoreductase 1
GCLM: Glutamate-cysteine ligase modifier subunit
TCGA: The Cancer Genome Atlas
CDS: Coding sequence
EGFR-TKI: Epidermal growth factor receptor tyrosine kinase inhibitor
EML4-ALK-TKI: Echinoderm microtubule-associated protein-like 4-anaplastic lymphoma kinase fusion tyrosine kinase inhibitor
ARE: Antioxidant responsive element
